# Functional Studies of Anodic Oxidized β-Ti-28Nb-11Ta-8Zr Alloy for Mechanical, *In-vitro* and Antibacterial Capability

**DOI:** 10.1038/s41598-018-32462-7

**Published:** 2018-09-24

**Authors:** Hsin-I Lin, Yu-Ming Kuo, Chun-Chih Hu, Mu-Huan Lee, Ling-Hsiang Chen, Chung-Tien Li, Tze-Hong Wong, Ta-Jen Yen

**Affiliations:** 1 Department of Materials Science and Engineering, National Tsing Hua University, Hsinchu, 30013 Taiwan; 2 Department of Orthopedics, National Taiwan University Hospital Hsinchu Branch, Hsinchu, 30059 Taiwan; 3 Center for Nanotechnology, Materials Science, and Microsystems, National Tsing Hua University, Hsinchu, 30013 Taiwan; 4 High Entropy Materials Center, National Tsing Hua University, Hsinchu, 30013 Taiwan

## Abstract

We developed an osseocompatible β-type Ti-28Nb-11Ta-8Zr (TNTZ) alloy that displays the excellent elastic modulus, cellular response, corrosion resistance and antibacterial capability demanded for bone-mimetic materials. The TNTZ alloy exhibited an elastic modulus of 49 GPa, which approximates that of human bones and prevent stress shielding effects. A further anodic oxidation and subsequent post-annealing modification formed a crystalline nanoporous TNTZ oxide layer (NPTNTZO(c)) on the alloy surface, potentially promoting interlocking with the extracellular matrix of bone cells and cell proliferation. Osteoblast viability tests also verified that NPTNTZO(c) enhanced cell growth more significantly than that of flat TNTZ. In addition, potentiodynamic polarization tests in Hanks’ balanced salt solution (HBSS) revealed that both TNTZ and NPTNTZO(c) exhibited better corrosion resistance than commercial pure titanium. Finally, NPTNTZO(c) reinforced with silver nanoparticles (NPTNTZO(c)/AgNPs) intensified the antibacterial efficiency against *Pseudomonas aeruginosa*, *Staphylococcus aureus* and *Escherichia coli* for 8 h with antibacterial efficiencies of 87.82%, 97.68%, and 93.90%, respectively, facilitating infection prevention during surgery and recovery stages.

## Introduction

With the increase in the occurrence of age-related degenerative diseases^1,2^ and traffic accidents^3–5^, there is now an increasing need for highly osseocompatible implant systems that enable rapid wound healing and sufficient osseointegration. Among currently available materials for medical applications, pure titanium and titanium-based alloys have emerged as primary candidates for bone implants because they possess modest mechanical, anticorrosive, and biocompatible properties^6–9^. Nevertheless, there is certainly demands for further improved pure titanium and titanium-based alloys to create excellent bone-mimetic materials. First, the commonly utilized commercial pure titanium (cp-Ti) and Ti-6Al-4V alloy (TAV) display elastic moduli of 100 and 112 GPa, respectively, exceeding those of natural bones (4–40 GPa)^6^. The mismatched elastic moduli between metal-based and biological materials easily augment the risk of the stress shielding effect, causing bone growth reduction, osteoporosis, and eventually bone tissue or implant deterioration^10,11^. To avoid the stress shielding effect without losing the implant gauges and to provide additional corrosion resistance, researchers have recently developed the quaternary alloy systems of Ti-29Nb-13Ta-4.6Zr and Ti-35Nb-7Ta-5Zr, which present the suitable elastic moduli of 65 and 55 GPa, respectively^7,12–16^.

Next, the biocompatibility of titanium and titanium-based alloys stems from the formation of a bioinert native oxide layer on their surfaces^17^. Unfortunately, this bioinert native oxide layer is usually amorphous, leading to relatively lower cell adhesion compared to that of the crystalline oxide surface^18^. In addition to achieving biocompatibility and comparable elastic moduli to natural bones, enhancing the osseointegration of the implants with the bones is another pivotal requirement of bone mimetics. Because bone tissues are composed of hierarchical nanostructured minerals^19–22^, introducing self-organized oxide nanotubes and/or nanopores on the surface of titanium implants fulfills this requirement^23–29^. Finally, implant-associated infections also create serious clinical issues. Once a severe infection occurs, patients would suffer from revision surgeries involving implant removal and re-implantation^24,30^. One proposed infection control measure entails covering the metal stem with a polymer membrane layer that slowly releases drugs (e.g., sirolimus or paclitaxel) to limit bacterial proliferation and restenosis^31^. However, inevitable polymer degradation may induce the inflammatory response, phagocytic activation, and vascular smooth muscle proliferation^31^. A suitable alternative would be modifying the implant surface with hydrophilic coatings that demote viral and microbial adhesion, thus preventing infection during and after surgery^32^.

To satisfy these aforementioned requirements of osseocompatible implants, we firstly introduced our own developed β-type Ti-28Nb-11Ta-8Zr (TNTZ) alloy, which displayed the excellent elastic modulus of 49 GPa, as the base alloy system in this study. This modulus is much closer to that of human bones; therefore, we can expect to avert the stress shielding effect. Additionally, we modified the surface of the TNTZ alloy via anodic oxidation (AO) and post-annealing heat treatments, forming a self-organized crystalline nanoporous TNTZ oxide layer (NPTNTZO(c)). Such a NPTNTZO(c) layer demonstrates high corrosion resistance^33^, protecting this alloy in the corrosive environment during osteoblast culture. Another advantage of NPTNTZO(c) is its nanoporous morphology^34^, which is biomimetic with the extracellular matrix of bone tissue, which is mainly composed of nanoporous minerals^35,36^. Furthermore, the NPTNTZO(c) layer can also act as a reservoir to accommodate silver nanoparticles (AgNPs), providing an improved antibacterial capability. The mechanical properties, chemical compositions, crystal structures, and corrosion resistance of TNTZ, NPTNTZO(c), and NPTNTZO(c)/AgNPs complex were determined to evaluate the optimal conditions that meet the criteria for potential bone implantation. Cellular responses, such as cell adhesion and viability, and antibacterial efficacy were investigated to define the conditions that simultaneously retard potential infection and promote cell viability and proliferation. The goal of this study is to evaluate the feasibility of our own developed β-type Ti-28Nb-11Ta-8Zr (TNTZ) alloy with the NPTNTZO(c)/AgNPs complex structure toward the application of orthopedic implantation.

## Results

### Mechanical properties of TNTZ alloy

**Figure 1:**
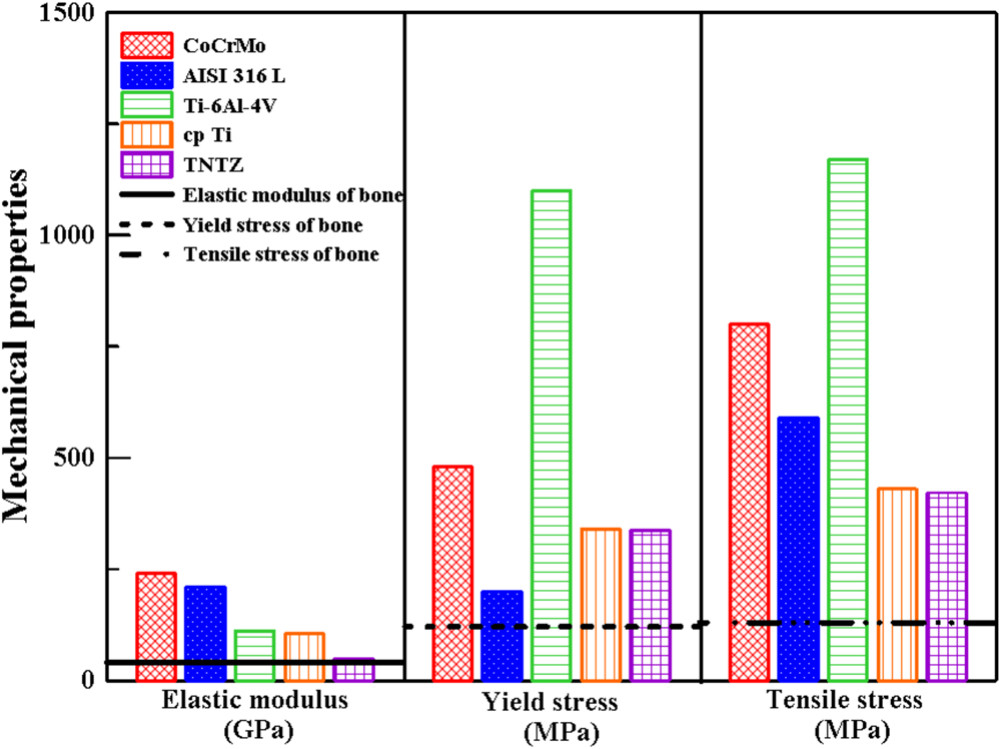
Comparisons of elastic modulus, yield stress, and tensile stress among CoCrMo, AISI 316 L stainless steel, Ti-6Al-4V, cp Ti, TNTZ and human bone.

The mechanical properties of TNTZ were, therefore, determined and systematically compared with those of the commonly applied medical implant materials: CoCrMo alloy, AISI 316 L stainless steel (316 L), TAV, and cp-Ti (Fig. 1). The elastic modulus is an important design requirement to prevent the stress shielding effect interfering bone remodeling in orthopedic implants. CoCrMo alloy (240 GPa), 316 L (210 GPa), TAV (112 GPa), and cp-Ti (105 GPa) exhibited higher elastic moduli than bone and TNTZ (49 GPa). This suggests that our TNTZ alloy behaves the most bone-mimic elastic modulus than the other alloy systems, shown in Fig. 1. Moreover, the yield strength (YS) and ultimate tensile strength (UTS) are also key mechanical properties that, unlike the elastic modulus, need to be maximized for implant applications. CoCrMo alloy, 316 L, TAV, cp-Ti, and TNTZ showed YS values of 480, 200, 1100, 340, and 338 MPa, respectively, and UTS values of 800, 590, 1170, 430, and 422 MPa, respectively (Fig. 1). To better describe the YS and UTS here, permissible strain, another significant design parameter for orthopedic implants defined as the ratio between YS and UTS, was applied^37^. This parameter should be larger than that for human cortical bone (0.67)^37^. Among these 5 alloy systems, TAV, cp-Ti and TNTZ are larger than criteria. Unlike high elastic-modulus TAV and cp-Ti, TNTZ, performed simultaneously with low elastic modulus and high permissible strain, is an ideal base-alloy model than the others.

### Microstructure and crystal structure of NPTNTZO/AgNPs

**Figure 2:**
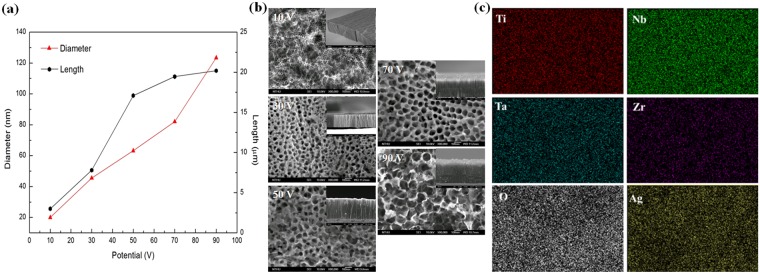
(**a**) Dimensional distributions of NPTNTZO(c) versus different applied voltages, (**b**) morphological observations of NPTNTZO(c) through top view and cross-section views (inset images) and (**c**) elemental mappings of NPTNTZO/AgNPs (scan area is 50 μm × 50 μm).

Because TNTZ displayed an elastic modulus that suppresses the stress shielding effect and a high permissible strain, the bone-mimetic porous NPTNTZO structure was synthesized on its surface by AO. This process enables the creation of porous and tubular microstructures separately and relies on open-circuit potential (OCP) to achieve control over the pore diameter of nanoporous oxide layers^38^. Therefore, the OCP was changed from 10 to 90 V to produce NPTNTZO layers with various pore diameters. Nanopore diameters and oxide layer thicknesses ranged from 20 to 125 nm and 3 to 20 µm, respectively (Fig. 2(a and b)). Because of size effects on cell culture^39,40^ and cavity size requirements for AgNP incorporation (10–30 nm in diameter), a nanoporous oxide layer with a diameter of 65 nm and thickness of 17 µm (Fig. 2(a and b)), fabricated at 50 V and 30 °C for 20 min in a water bath, was chosen for the remaining experiments. Next, the NPTNTZO layer was transformed into NPTNTZO(c) by heat treatment.

**Figure 3:**
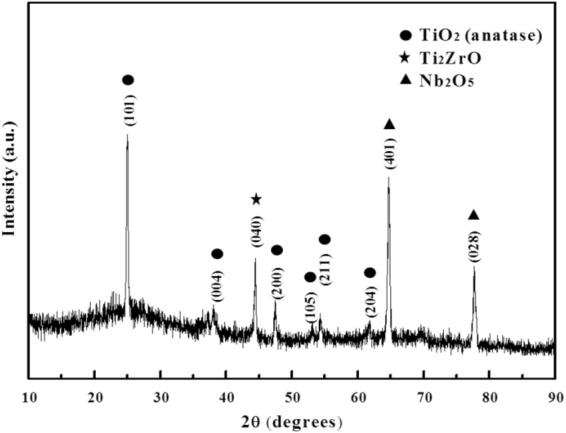
XRD pattern of NPTNTZO after post-annealing.

The surface chemical compositions of AgNP-containing NPTNTZO(c) layers were determined by energy dispersive spectroscopy (Fig. 2(c)). Elemental mapping of Ti, Nb, O, Ta, Zr, and Ag signals showed that the nanoporous oxide layer homogeneously covered the alloy surface and AgNPs were also well distributed over this oxide layer. This uniform NPTNTZO(c)/AgNPs structure is expected to remain fully intact before and after surgery. XRD pattern of NPTNTZO(c) after annealing at 700 °C indicated that the significant crystalline metal oxides formed as anatase TiO_2_ (100), orthorhombic Ti_2_ZrO, and orthorhombic Nb_2_O_5_ phases (Fig. 3).

### Chemical compositions of NPTNTZO/AgNP

**Figure 4:**
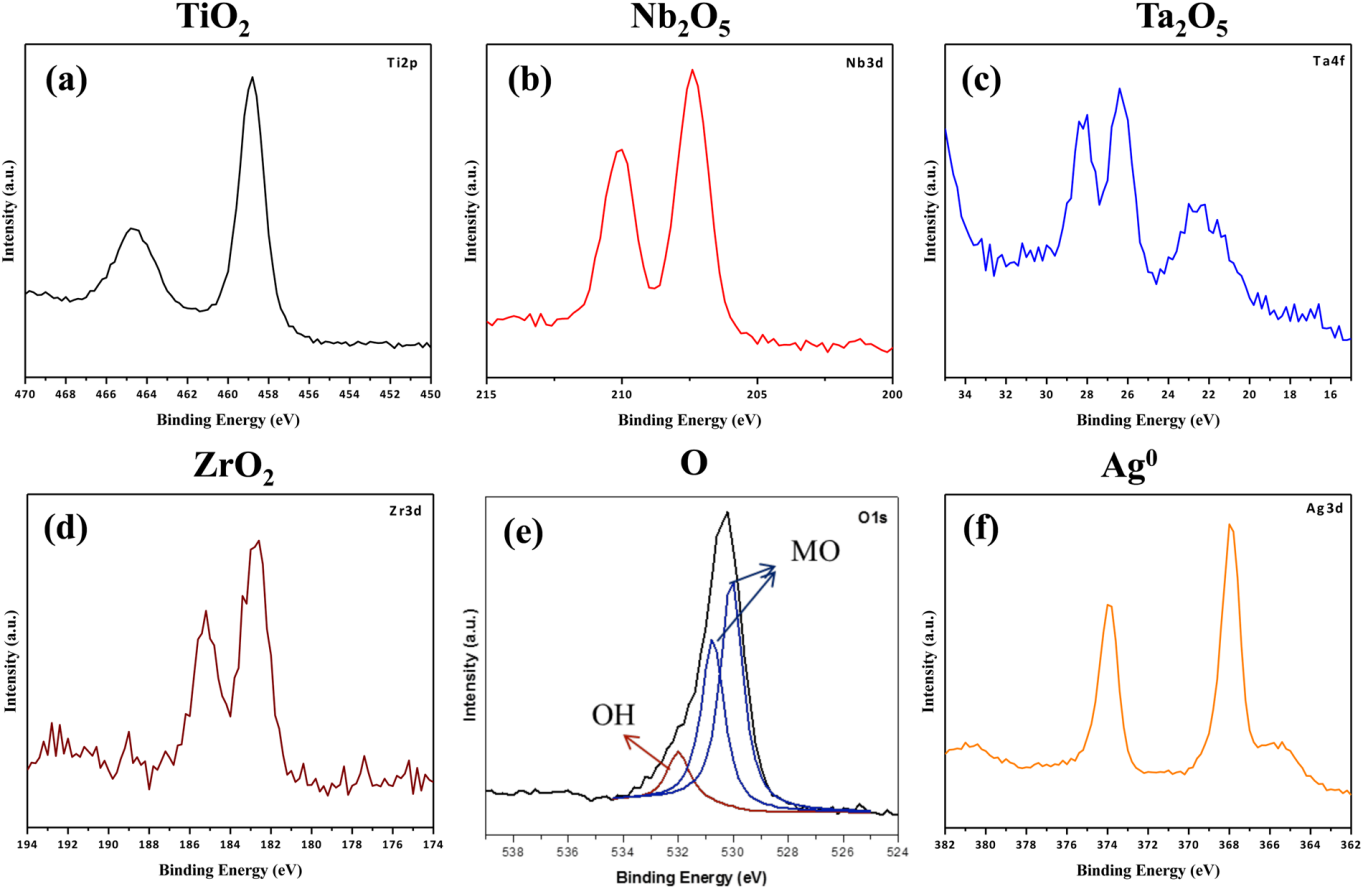
High resolution XPS results of (**a**) TiO_2_ (Ti 2p), (**b**) Nb_2_O_5_ (Nb 3d), (**c**) Ta_2_O_5_ (Ta 4f), (**d**) ZrO_2_ (Zr 3d), (**e**) O (O 1s) and (**f**) Ag° (Ag 3d) with C1s peak as calibration.

**Figure 5:**
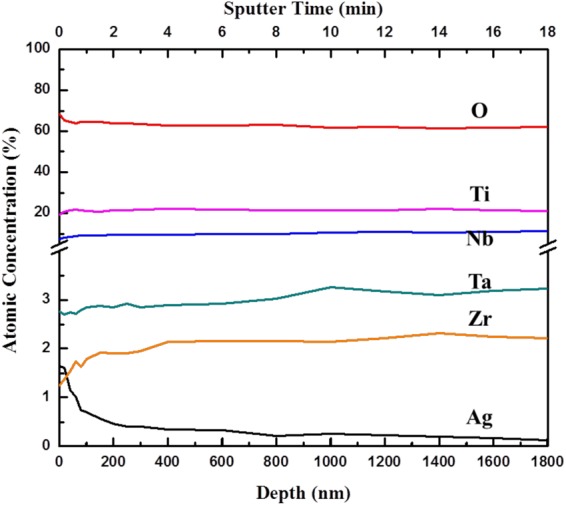
Atomic concentration distributions of Ti, Ta, Nb, Zr, O and Ag on NPTNTZO(c)/AgNPs sample through XPS depth profiles.

Elemental distributions of NPTNTZO(c)/AgNPs (Fig. 2(c)) demonstrated that AgNPs were uniformly distributed on the NPTNTZO(c) layer. Precise and quantitative distributions of chemical compositions of NPTNTZO(c)/AgNPs were acquired by XPS (Fig. 4) and depth profiles (Fig. 5). All XPS binding energies were calibrated using the peak corresponding to the C 1 s orbital at 284.8 eV. High-resolution spectra for Ti 2p, Nb 3d, Ta 4f, Zr 3d, O 1 s, and Ag 3d are shown in Fig. 4. The XPS spectrum of Ti 2p exhibited characteristic peaks for Ti 2p_3/2_ and Ti 2p_1/2_ at 458.8 and 464.8 eV, respectively (Fig. 4(a)), consistent with the presence of TiO_2_. Figure 4(b) showed two characteristic peaks at 207.2 and 210 eV corresponding to Nb 3d_5/2_ and Nb 3d_3/2_, respectively, indicating the formation of Nb_2_O_5_. The spectrum of Ta 4 f displayed one peak at 23 eV for Ta 4f_7/2_, suggesting the existence of pure Ta, and peaks at 26.4 eV for Ta 4 f as well as at 28 and 28.4 eV Ta 4f_5/2_, indicating the presence of Ta_2_O_5_ (Fig. 4(c)). As shown in Fig. 4(d), the spectrum of Zr 3d presented binding energies at 182.6 and 185.2 eV corresponding to Zr 3d_5/2_ and Zr 3d_3/2_, respectively, demonstrating the formation of ZrO_2_.

The spectrum of O 1 s (Fig. 4(e)) was fitted with three asymmetric Gaussian curves, revealing two fitted Gaussian peaks at 530 and 530.8 eV representing the lattice oxygen in mixed metal oxides (MO, M = Ti, Nb, Ta, and Zr) and one peak at 531.9 eV corresponding to the surface hydroxyl groups (-OH). In summary, XPS spectra for Ti 2p, Nb 3d, Ta 4f, Zr 3d, and O 1 s (Fig. 4(a–e)) confirmed the presence of TiO_2_, Nb_2_O_5_, Ta_2_O_5_, and ZrO_2_ in NPTNTZO(c)/AgNPs. The XPS spectrum of Ag 3d shown in Fig. 4(f) exhibited peaks at 368 and 374 eV, which represented Ag 3d_5/2_ and Ag 3d_3/2_ in Ag^0^, consistent with the existence of AgNPs.

### Corrosion resistance

**Figure 6:**
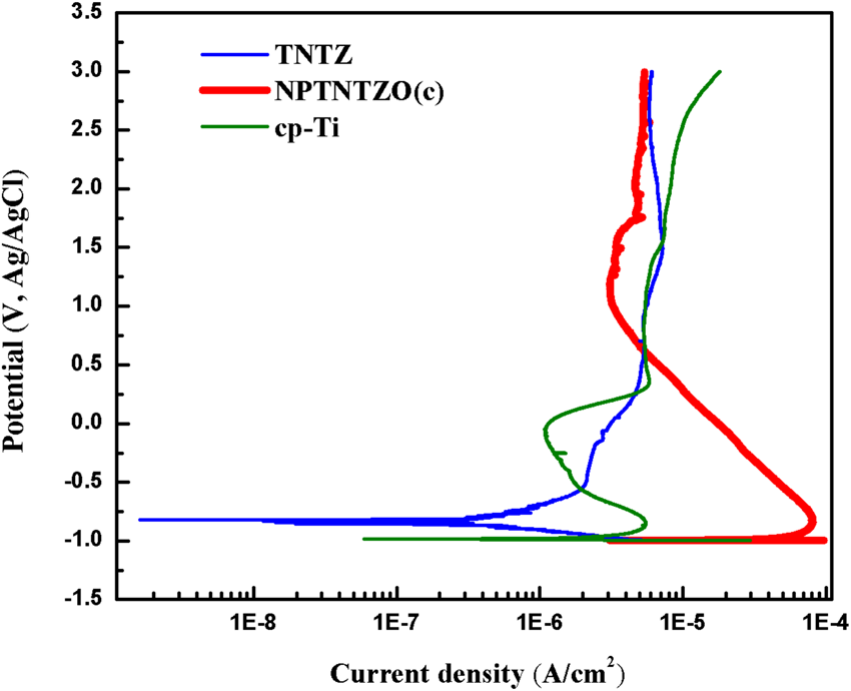
Potentiodynamic polarization curves of TNTZ, NPTNTZO(c) and cp-Ti in HBSS at 37 °C.

Potentiodynamic polarization tests were conducted to compare the corrosion resistance of TNTZ, NPTNTZO(c), and cp-Ti in HBSS. TNTZ exhibited the highest corrosion potential *E*_corr_ (−836 mV) and the lowest corrosion current *I*_corr_ (0.32 µA cm^−2^) compared with NPTNTZO(c) and cp-Ti, indicating its greater corrosion resistance in HBSS in the early stage of corrosion (Fig. 6 and Table 1). Interestingly, TNTZ showed long-range passivation instead of a noticeable breakdown potential (Fig. 6). Comparable passivation currents (*I*_pass_) were observed for TNTZ (2.45 µA cm^−2^), NPTNTZO(c) (3.1 µA cm^−2^), and cp-Ti (1.09 µA cm^−2^). Also, crossing voltages of 2,003 and 773 mV were obtained for NPTNTZO(c) and cp-Ti, respectively. This suggests NPTNTZO(c) presents the stronger corrosion resistance in the late stage of corrosion.

**Table 1:** Corrosion potential, current density, breakdown potential, passive current and crossing voltage of TNTZ, NPTNTZO(c) and cp-Ti in HBSS at 37 °C.

Sample	*E*_corr_ (mV)	*I*_corr_ (*µ*A cm^−2^)	*E*_break_ (*mV*)	*I*_pass_ (µA cm^−2^)	Crossing Voltage (mV)
Flat TNTZ	−836	0.32	—	2.45	—
NPTNTZO	−996	23.4	1700	3.1	2003
cp-Ti	−986	1.3	−9	1.09	773

### Cell proliferation and viability

**Figure 7:**
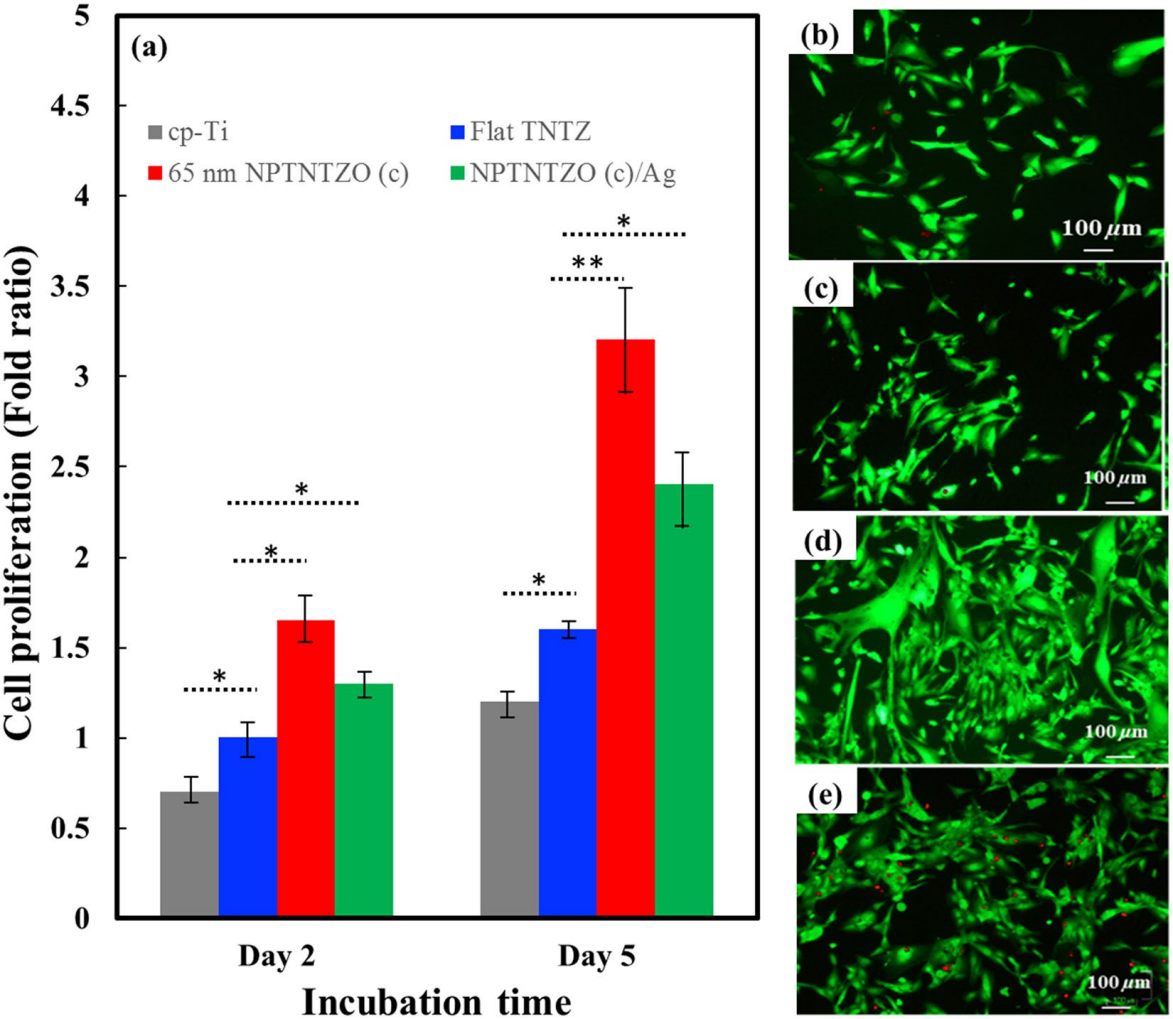
(**a**) hFOB proliferation on cp-Ti, TNTZ, 65 nm NPTNTZO(c) and NPTNTZO(c)/1.62 at.% AgNPs for 2 days and 5 days incubation. The data are repeated 3 times. N = 3 (T-test is utilized to determine the level of significance. *p < 0.05, and **p < 0.001. Live/Dead cell viability was shown in (**b)** cp-Ti, (**c**) TNTZ, and (**d**) 65 nm NPTNTZO(c) and (**e**) NPTNTZO(**c**)/1.62 at.% AgNPs for 2 days incubation.

The proliferation of hFOB on cp-Ti, TNTZ, 65 nm NPTNTZO(c), and NPTNTZO(c)/1.62 at.% AgNPs were analyzed by MTT colorimetric assay. Statistical results of the MTT assay (Fig. 7(a)) provide information that the hFOB exhibited significantly higher cell proliferation on 65 nm NPTNTZO(c) than on cp-Ti and TNTZ after two- and five-day incubations because the crystalline oxide layers resulted in more exposed hydroxyl groups on the surfaces^41^. In addition, a drop in hFOB density observed on NPTNTZO(c)/1.62 at.% AgNPs compared with 65 nm NPTNTZO(c), indicating that AgNPs could inhibit cell growth^42^. Live/dead staining assays (Fig. 7(b–e)) gave additional data on hFOB viability after two-day incubation. More live cells were detected by green fluorescence on 65 nm NPTNTZO(c) and NPTNTZO(c)/1.62 at.% AgNPs than on cp-Ti and TNTZ. However, more dead cells upon staining using the red fluorescent dye were also found on NPTNTZO(c)/1.62 at.% AgNPs than on 65 nm NPTNTZO(c), suggesting that the existence of AgNPs may inhibit cell growth.

### Antibacterial efficacy

**Figure 8:**
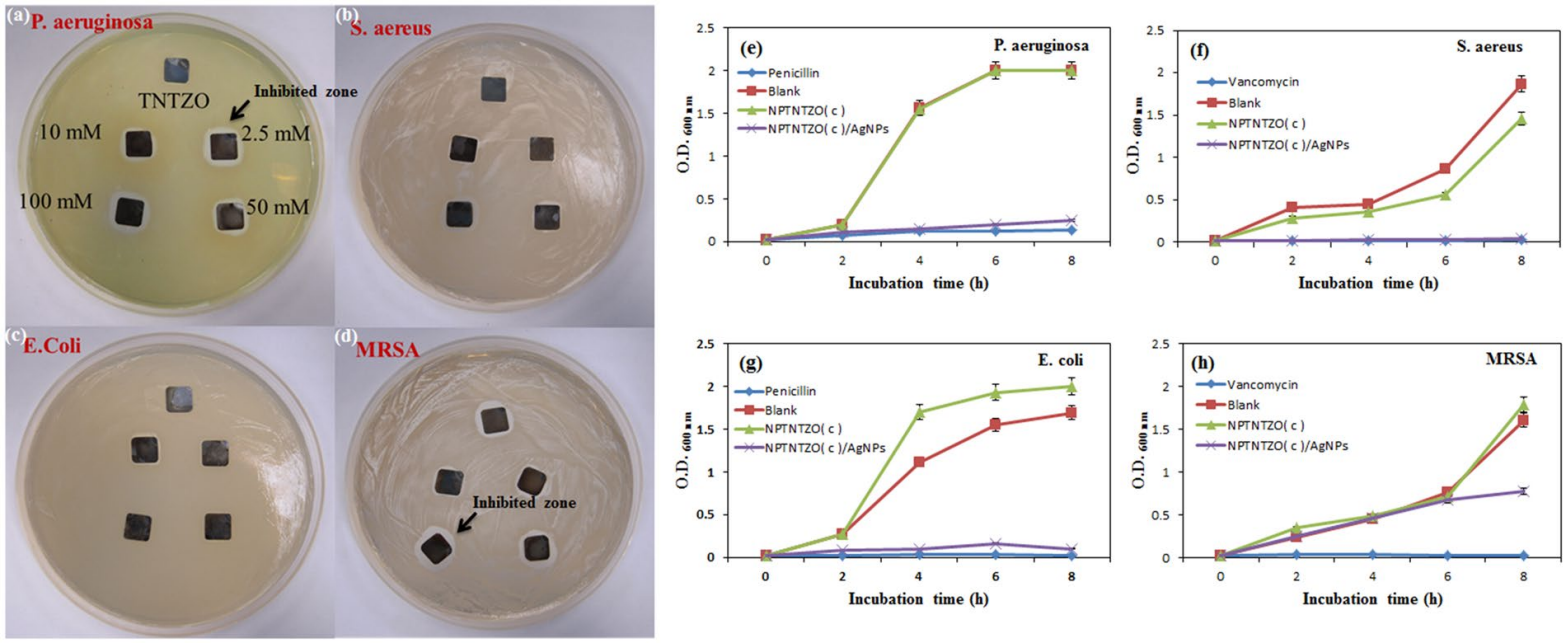
(**a**), (**b**), (**c**) and (**d**) reveal the inhibited zones formed on NPTNTZO with various concentrations of AgNPs under *P. aeruginosa*, *S. aereus*, *E. coli* and MRSA solution, respectively. Historical bacterial growth curves of (**e**) *P. aeruginosa*, (**f**) *S. aereus*, (**g**) *E. coli* and (**h**) MRSA among antibiotics, NPTNTZO, NPTNTZO/AgNPs, and pure bacteria solution (blank group).

The qualitative variations in antibacterial efficacy of five different AgNP dosages (0, 2.5, 10, 50, and 100 mM AgNO_3_ solutions) were evaluated by the Kirby–Bauer test^43^. For all AgNP dosages, the inhibited zones presented almost identical areas, making discrepancies difficult to distinguish. The inhibited zones of *P. aeruginosa*, *S. aureus*, *E*. *coli* and MRSA against NPTNTZO(c)/1.62 at.% AgNPs are shown in Fig. 8(a–d), respectively.

Next, the growth curves of pure *P. aeruginosa*, *S. aureus*, *E*. *coli* and MRSA bacterial solutions on antibiotics (Penicillin and Vancomycin), NPTNTZO(c) and NPTNTZO(c)/1.62 at.% AgNPs were monitored quantitatively (Fig. 8(e–h)). Growth curves of pure bacterial solutions were also determined as controls. The growth curves first displayed a lag phase, followed by an exponential growth phase, and ended with a stationary phase. The starting point of this stationary phase is an important index of antibacterial efficiency. *P. aeruginosa* reached the stationary phase after 6 h on NPTNTZO(c) (Fig. 8(e)), consistent with the time obtained for the bacterial solution. In contrast, the beginning of the stationary phase was delayed to more than 8 h on NPTNTZO(c)/1.62 at.% AgNPs. Similar results were obtained for *S. aureus* (Fig. 8(f)). NPTNTZO(c)/1.62 at.% AgNP delayed the stationary phase to more than 8 h compared with those of NPTNTZO(c) (8 h) and pure *S. aureus* solution. For *E. coli* (Fig. 8(g)), the stationary phase was delayed to more than 8 h on NPTNTZO(c)/1.62 at.% AgNPs compared with those of NPTNTZO(c) (6 h) and pure *E. coli* solution. However, MRSA (Fig. 8(h)) exhibited the higher OD value on NPTNTZO(c)/1.62 at.% AgNPs.

Serial dilution and spread-plate methods were conducted at 14 h of growth to determine the colony-forming units of *P. aeruginosa* and *S. aureus*, *E*. *coli* and MRSA. The CFU values at 8 h (Table 2) showed that bacterial concentrations on NPTNTZO(c) were smaller than in solution because of the hydrophilicity of oxide nanostructure surfaces^44–46^. NPTNTZO(c)/1.62 at.% AgNPs further limited bacterial growth because of the additional antibacterial agent. Penicillin and Vancomycin exhibited highly antibacterial efficiencies to *P. aeruginosa* and *S. aureus*, *E*. *coli* and MRSA with efficiencies of 93.27%, 98.52%, 99.02% and 98.38%, respectively. The antibacterial efficiencies of the modified surfaces at 14 h (Table 2) revealed that NPTNTZO(c)/1.62 at.% AgNPs reduced *P. aeruginosa* and *S. aureus*, *E*. *coli* and MRSA strains with efficiencies of 87.82%, 97.68%, 93.90%, and 52.07%, respectively. In contrast, NPTNTZO(c) exhibited the efficiencies of only 22.01% against *S. aureus*.

**Table 2:** Antibacterial efficiency of the modified surfaces under 14^th^ h incubation.

Group (8^th^ h)	O.D. value (600 nm)	Antibacterial efficiency (%)
P.A._Pure_	2	—
P.A._Penicillin_	0.13	93.27
P.A._NPTNTZO_	2.00	0
P.A._NPTNTZO/AgNPs_	0.24	87.82
E. coli_Pure_	1.69	—
E. coli _Penicillin_	0.02	99.02
E. coli _NPTNTZO_	2.00	0
E. coli_NPTNTZO/AgNPs_	0.10	93.90
MRSA_Pure_	1.61	—
MRSA_Vancomycin_	0.03	98.38
MRSA_NPTNTZO_	1.79	0
MRSA_NPTNTZO/AgNPs_	0.77	52.07
S.A._Pure_	1.87	—
S.A._Vancomycin_	0.03	98.52
S.A._NPTNTZO_	1.46	22.01
S.A._NPTNTZO/AgNPs_	0.04	97.68

## Discussion

The original mechanical properties of stem materials play the first and significant factors to be considered when determining an ideal bone implant. We separated into two categories: elastic modulus and permissible strain. The elastic moduli of CoCrMo alloy, 316 L, TAV, and cp-Ti are obviously much greater than that of bone tissue, resulting in a deficient loading on the adjacent remodeled bone, osteoporosis, and the breakdown of bone tissues or implants^10,11,47^. In contrast to this, this self-synthesized β-type TNTZ displayed a bone-similar elastic modulus of 49 GPa. This is attributed to the following two major factors: (1) the addition of β stabilizers (Nb, Ta) that decrease the modulus of body-centered cubic Ti without weakening, and (2) the addition of the neutral element Zr that retards martensitic transformation during cooling^48^. Therefore, the β-type Ti alloy is more beneficial for implant applications than its α and (α + β) counterparts^6^. Interestingly, the elastic modulus of Ti-28Nb-11Ta-8Zr was lower than those of Ti-29Nb-13Ta-4.6Zr (65 GPa) and Ti-35Nb-5Ta-5Zr (55 GPa)^6,33^ owing to its higher Zr content. Although Zr is not a β stabilizer, a high Zr content lowered the elastic modulus of Ti to a similar extent as would be the case when using β stabilizers. In particular, 6% Zr generated the same effect as 1.5% Mo^49^.

Besides the elastic moduli to these alloy systems, the calculated permissible strain values amounted to 0.60, 0.34, 0.94, 0.79, and 0.80 for CoCrMo alloy, 316 L, TAV, cp-Ti, and TNTZ, respectively. These YS, UTS, and permissible strain results suggest that TAV may be the optimal candidate for bone implants (larger than 0.67). However, the presence of toxic aluminum and vanadium elements in its composition and its high elastic modulus limit its application in clinical implantation. However, TNTZ free from these toxic and allergenic elements, presented a bone-like elastic modulus and moderate permissible strain, making it suitable for clinical use. These results also indicate that Ti-28Nb-11Ta-8Zr alloy can perfectly mimic the mechanical properties of bone tissue more so than other medical implant alloys.

We initiated the AO process and post-heat treatment on TNTZ foils to create NPTNTZO(c) layer. Two significant crystalline metal oxides, Anatase TiO_2_ and orthorombic Nb_2_O_5_, of NPTNTZO(c) are two significant crystalline metal oxide that are beneficial to antibacterial efficacy and cellular activities. Anatase TiO_2_ and orthorombic Nb_2_O_5_ both are expected to provide antibacterial resistance and hydrophillicity because of their photocatalytic property^50–54^. These properties can be further enhanced under sufficient exposure to light^50,55^. Anatase TiO_2_ also increase mechanical properties, such as abrasive wear resistance and corrosion resistance^56^. In order to further support antibacterial efficacy, we took advantage of the 65-nm-width nanoporous structure after the AO process to load AgNPs for a long-term activation. Next, the XPS data discovered the distribution of AgNPs and the contents of TiO_2_, Nb_2_O_5_, Ta_2_O_5_, and ZrO_2_ in NPTNTZO(c)/AgNPs. The depth profile of NPTNTZO(c)/AgNPs decreased dramatically in the presence of 2.5 mM AgNP solution (Fig. 5), thus suggesting that AgNPs mostly accumulated on the top surface of the nanoporous oxide layer. The concentration of AgNPs on the surface amounted to 1.62 at.% (2.5 mM AgNO_3_ solution) and the distribution depth reached 600 nm, indicating that AgNPs were successfully incorporated into the nanopores by photoreduction.

To estimate the lifetime of the implant, potentiodynamic polarization presents a great methodology. Among the indices (corrosion potential *E*_corr_, corrosion current *I*_corr_, breakdown potential *E*_break_, passivation currents *I*_pass_ and Crossing voltage), TNTZ suggests its greater corrosion resistance in HBSS than NPTNTZO(c) and cp-Ti. NPTNTZO(c) (*E*_corr_ = −996 mV; *I*_corr_ = 23.4 µA cm^−2^) exhibited a higher corrosion rate than TNTZ and cp-Ti. Specifically, it formed a passive layer and displayed a slight breakdown at 1700 mV, which was followed by a second passivation with a small increase in current density. The higher corrosion rate of NPTNTZO(c) stemmed from the larger specific surface area of the nanoporous surface^57,58^. Immediately afterward, the passive layer formed inside the NPTNTZO(c) nanopores, enhancing corrosion resistance^59,60^. cp-Ti showed a clear breakdown potential at −9 mV and a second passivation when the current density slightly increased after the breakdown. The high crossing voltage of NPTNTZO(c) presented higher corrosion resistance (lower corrosion rate) in the passive region than cp-Ti. In summary, although NPTNTZO(c) showed a lower passivation range than TNTZ, it demonstrated a better performance than cp-Ti. Importantly, because of its high corrosion-resistant potential in HBSS since passive region, NPTNTZO(c) has potential to be used for long-term implantation within the corrosive environment of simulated body fluid.

The results of cell proliferation and viability indicated that 65 nm NPTNTZO(c) actually promoted cell proliferation and viability more significantly than on cp-Ti and flat TNTZ. However, the addition of AgNPs, which was slowly released from oxide layer cavities during cell culture periods, retarded cell growth. AgNPs indeed inhibited cell growth during the initial cell culture period (two-day incubation) but hFOB adapted the microenvironment from NPTNTZO(c)/1.62 at.% AgNPs to induce cell growth for a longer culture period (five-day incubation). Consequently, with careful control of the AgNPs concentration, NPTNTZO(c)/AgNPs implants can simultaneously maintain mammalian cells and achieve the desired antimicrobial level^43^. Previous studies have also suggested that surface modification of AgNPs using glucose, lactose, and oligonucleotides^61,62^ may decrease their cytotoxicity and offer new therapeutic applications toward clinical cancer diagnosis and treatment.

From the results evaluated via Kirby–Bauer test, *P. aeruginosa* and MRSA displayed a slightly larger inhibited zone than *S. aureus* and *E. coli*. This difference may stem from bacterial mobility, continuous Ag^+^ ion leaching, and hydrophilic surface. Initially, *P. aeruginosa* and MRSA showed higher mobility than *S. aureus* and *E. coli* because of its flagellum^63–65^. The highly mobile *P. aeruginosa* and MRSA were continuously contacted and attacked by the leaching Ag^+^ ions, causing its inhibited zone to expand beyond that obtained by *S. aureus* and *E. coli*. S. Kim *et al*.^66^ studied the manner in which bacteria repelled from highly-ordered alumina nanopore structure and confirmed that mechanism of bacterial adhesion depends on cell-surface contact area and water-substrate contact angle. This explains the reason for which our hydrophilic NPTNTZO surface provided more favorable attractive forces that promoted the absorption of bacteria into the nanopores, where the bacteria perished quickly because of poor nutrition inside the porous structure. When AgNPs is incorporated into such a surface, we can see that halo of inhibition is more obvious on P. aerugunosa and MRSA. Consequently, clear inhibited zones were obtained at 1.62 at.% AgNPs.

From the growth curves of the bacterial solution, we obtained the stationary time and OD numbers among tested samples. NPTNTZO(c)/1.62 at.% AgNPs presented similar stationary time (6–8 h) and a lower optical density against all bacterial solutions, expect MRSA. This indicates that NPTNTZO(c)/1.62 at.% AgNPs sufficicently inactivated the growth of *P. aeruginosa S. aureus* and *E*. *coli*; however, Ag ion poorly inhibit the growth of MRSA. This result is due to the dilayer bacteria capsule preventing the demolishing from the Ag ion. Remarkably, *P. aeruginosa* exhibited longer resistance against the Ag environment than *S. aureus* because its cell walls could trap and detoxify Ag^+^ ions before dying^66^. However, NPTNTZO(c)/1.62 at.% AgNPs is not capable of providing significant inhibition on MRSA. This might be explained that the less effective biomass reduction of MRSA from AgNPs^67^.

## Conclusions

Our own developed Ti-28Ta-11Nb-8Zr (TNTZ) alloy system exhibited a bone-mimetic elastic modulus of 49 GPa, preventing the stress shielding effect that commonly appears in the commercial cp-Ti and other implants, as well as the ideal permissible strain. This proves TNTZ alloy system applied in this study promise it feasibility of being orthopedic joint from the mechanical point of view. A self-organized crystalline nanoporous TNTZ oxide layer (NPTNTZO(c)) showed a high corrosion resistance with the low passive current density to ensure long-term implantation. In addition, *in vitro* cell viability and proliferation assessments suggested that both NPTNTZO(c) and NPTNTZO(c)/AgNPs contributed to cell viability. Moreover, the NPTNTZO(c) nanoporous structure acted as a reservoir for AgNPs, leading to an antibacterial efficiency reaching 87.82%, 97.68%, and 93.90% against *P. aeruginosa* and *S. aureus*, and *E*. *coli*, respectively. These excellent properties demonstrate that TNTZ/NPTNTZO(c)/AgNPs implant systems provide a ready solution for osseocompatible materials beyond current commercial implants.

## Materials and Methods

### Fabrication of TNTZ and NPTNTZO/AgNPs

β-type Ti-28Nb-11Ta-8Zr (TNTZ) alloy was fabricated in an arc melting furnace with a non-consumable tungsten electrode and a water-cooled copper crucible under Ar atmosphere. To homogenize the alloy, all the ingots were melted, solidified, inverted and then re-melted for 5 times. Afterwards, ingots were forged and then sliced by a wire-cutting technique into foils with the size of 10 mm × 10 mm × 1 mm. The TNTZ foils were solutionized at 1063 K in Ar gas atmosphere for 1 h, and quenched in water to stabilize β phase of TNTZ. Then, these foils were sequentially ground with emery papers up to #4000, followed by ultrasonication in ethanol, acetone and deionized (DI) water for 30 min, and dried in N_2_ stream.

Next, a conventional two-electrode setup utilized in AO process was applied ethylene glycol solution (0.6 wt% ammonium fluoride (NH_4_F) and 3 vol% DI water) as electrolyte to form a continuous layer of NPTNTZO on TNTZ foils under 50 V and 30 °C for 20 min in water bath. Then, the synthesized amorphous NPTNTZO was transformed into NPTNTZO(c) after post heat treatment (700 °C in Ar atmosphere for 1 h). Finally, we soaked NPTNTZO(c) samples within AgNO_3_ solutions under the concentrations of 2.5 mM, 10 mM, 50 mM and 100 mM for 10 min, respectively, and the AgNPs after photo-reduction process were loaded into the nanoporous structures.

### Mechanical properties of β-type Ti-28Nb-11Ta-8Zr

The long bars of TNTZ ingots for tension specimen were cut by a wire-cutting technique^68^. Young’s modulus, yield stress, and tensile stress of the prepared TNTZ samples were evaluated by a universal testing machine.

### Microstructural characterizations and compositional examinations

The morphology of NPTNTZO(c)/AgNPs was characterized by a field-emission scanning electron microscope (FESEM, JSM-7000F), operating at 10 KV. Crystal structure of NPTNTZO(c) was identified by a thin-film x-ray diffractometer (XRD, RU-H3R) with the scanning angle ranging from 10° to 90° at the scan rate of 4°/min. The chemical compositions were analyzed by an x-ray photoelectron spectroscope (XPS, ULVAC-PHI), with an x-ray source of an Al anode operated at 3 kV and a take-off angle of 45°. The covalent charges of the component of NPTNTZO(c)/AgNPs were investigated by the binding energy of Ti 2p, Nb 3d, Ta 4 f, Zr 3d and O 1 s with C 1 s (i.e. 284.8 eV) as references. Besides, the depth profile of XPS was also plotted for probing the distribution of Ag 3d along the depth of nanopores. The peaks were curve-fitted and deconvoluted by the XPSpeak41 software.

### Electrochemical analysis

To compare the corrosion resistance among TNTZ, NPTNTZO(c), and cp-Ti, all samples were cold mounted within epoxy resin. The exposed test surface (area: 1 cm^2^) was ultrasonically rinsed in ethanol and DI water for 10 min before the test. The Hanks’ balanced salt solution (HBSS), a simulated body fluid solution, (Biowest, ingredient: 0.137 M NaCl, 5.4 mM KCl, 0.25 mM Na_2_HPO_4_, 0.44 mM KH_2_PO_4_, 1.3 mM CaCl_2_, 1.0 mM MgSO_4_ and 4.2 mM NaHCO_3_) was used as the electrolyte and maintained at the pH value of 7.4 and the temperature of 37 °C during the electrochemical analysis. Potentiodynamic polarization was measured by a computer-controlled potentiostat (CHI 600 A; software: Electrochemical analyzer, CHI604A), in which Ag/AgCl with saturated KCl functions as the reference electrode, Pt foil as the counter electrode, and the test material as the working electrode. The potential applied to the working electrode ranged from −1000 mV to 3000 mV at the scan rate of 1 mVs^−1^.

### ***In-vitro*** cell proliferation and viability tests

A human fetal osteoblast cell line (hFOB 1.19, ATCC, CRL-11372) was cultured in a 90% Dulbecco’s modified eagle’s medium (DMEM)- rich glucose with L-glutamine, sodium pyruvate (biowest), 1% penecilin-streptomycin (Sigma-Aldrich) and 10% fetal bovine serum (FBS, SAFC_Biosciences_) at 33.5 °C and 5% CO_2_ and was ready to be cultivated on our testing samples. Meanwhile, all testing samples (Flat TNTZ, TNTZO(c) and crystal TNTZO(c)/AgNPs) were sterilized by autoclave (125 °C, 15 psi, 15 min) prior to the following *in-vitro* cellular and antibacterial tests.

For cell proliferation, all samples were placed in the 24-well plate to be cultivated with 4.8 × 10^5^ hFOB cells/mL for two days and five days. hFOB cells cultivated samples after two- and five-days incubation were first rinsed with PBS and transferred to a new 24-well plate and 300 mL MTT assay (3-(4,5-dimethylthiazole-2-yl)-2,5-diphenyl tetrazolium bromide, Sigma-Aldrich) was subsequently added to each well for another 4 h incubation at CO_2_ incubator (37 °C and 5% CO_2_). Dimethyl sulfoxide (DMSO, Sigma-Aldrich) was thereafter added to dissolve formazan and gently shaken the 24-well plate for 15 min. Finally, the ELISA (enzyme linked immunosorbent assay) reader was recorded the absorbance of the hFOB-cultivated samples (Chromate microplate reader).

For cell viability, the Live/Dead staining assay was performed to evalute the viability of hFOB. 10^6^ cells/mL hFOB cells were seeded on our samples placed in 35 mm Petri dishes, and incubated them for two and five days in CO_2_ incubator (37 °C and 5% CO_2_)_._ After incubation, Calcein-AM solution (2 μM in PBS) and ethidium homodimer-1 solution (EthD-1, 4 μM in PBS) were added onto the hFOB-cultivated samples for 30 min at room temperature for staining the Live/Dead cells. Then, hFOB-cultivated samples were rinsed with PBS twice, followed by analyzing the cell viability by using the LIVE/DEAD Viability/Cytotoxicity Kit (Molecular Probes, L-3224). The live and dead hFOB cells were stained in green and red accordingly and imaged by a fluorescence microscope (Nikon 50I).

### Bacterial incubation and antibacterial assay

*P. aeruginosa*, *S. aureus*, *E*. *coli* and MRSA (isolated from National Taiwan University Hospital Hsinchu Branch) were cultivated in the BBL Trypticase Soy Broth (Soybean-casein Digest Broth) at 37 °C for 18 h. Inhibited zone observation was conducted by the qualitative Kirby-Bauer test. *P. aeruginosa*, *S. aureus*, *E*. *coli* and MRSA solutions with the initial concentration of 10^7^ CFU/ ml (Optical intensity (OD) value of 0.25) were separately dropped and uniformly spread onto the agar plates (Mueller Hinton II Agar). A variety of NPTNTZO(c) and NPTNTZO(c)/AgNPs chips loaded with different AgNO_3_ concentrations (2.5, 10, 50, and 100 mM) were contacted with the bacteria-coated agar plates at 37 °C for 24 h for the following inhibited zone observation.

Positive control groups are selected Penicillin and Vancomycin, well known antibiotics for Gram-negative (*P. aeruginosa* and *E*. *coli*) and Gram-positive (*S. aureus* and MRSA) bacteria, respectively. As for the antibacterial efficiency test, antibiotics, NPTNTZO(c) and NPTNTZO(c)/AgNPs were immersed in *P. aeruginosa*, *S. aureus*, *E*. *coli* and MRSA solutions with the initial concentration of 10^6^ CFU/ ml (OD value of 0.08), respectively. OD values of antibiotics, NPTNTZO(c) and NPTNTZO(c)/AgNPs cultivated with pure *P. aeruginosa*, *S. aureus*, *E*. *coli* and MRSA solutions were recorded during the 8-hour growing period. Bacterial solutions at 8^th^ h were counted by a serial culture method accompanied with OD recording to retrieve the historical growth curve of bacteria. All the tests were repeated three times.

## References

[1] Mamaril ME, Childs SG, Sortman S (2007). Care of the orthopaedic trauma patient. J Perianesth Nurs.

[2] Cheng CW, Solorio LD, Alsberg E (2014). Decellularized tissue and cell-derived extracellular matrices as scaffolds for orthopaedic tissue engineering. Biotechnol Adv.

[3] Abdel-Hady Gepreel M, Niinomi M (2013). Biocompatibility of Ti-alloys for long-term implantation. J Mech Behav Biomed Mater.

[4] Delaunay CP (2004). Metal-on-metal bearings in cementless primary total hip arthroplasty. J Arthroplasty.

[5] Nabeel, S. Scientific Editorial-Combined Endodontic-Periodontic-Orthodontic-interdisciplinary treatment aroach in a periodontally compromised maxillary central incisor-A Case Presentation. *Dental Follicle -The E-Journal Of Dentistry* 85–87 (2012).

[6] Geetha M, Singh AK, Asokamani R, Gogia AK (2009). Ti based biomaterials, the ultimate choice for orthopaedic implants – A review. Prog. Mater Sci..

[7] Mohammed, M. T., Khan, Z. A. & Siddiquee, A. N. Beta titanium alloys: the lowest elastic modulus for biomedical applications: a review. *Int. J. Chem. Mol. Nucl. Mater. Metall. Eng*. **8** (2014).

[8] Niinomi M, Nakai M, Hieda J (2012). Development of new metallic alloys for biomedical applications. Acta Biomater.

[9] Wei Q (2011). Influence of oxygen content on microstructure and mechanical properties of Ti–Nb–Ta–Zr alloy. Materials & Design.

[10] Meijerink HJ, van Loon CJ, de Waal Malefijt MC, van Kampen A, Verdonschot N (2010). A sliding stem in revision total knee arthroplasty provides stability and reduces stress shielding. Acta Orthop.

[11] Engh, C. A. Jr., Young, A. M., Engh, C. A. Sr. & H, R. H. Jr. Clinical consequences of stress shielding after porous-coated total hip arthroplasty. *Clin Orthop Relat Res*, 157–163 (2003). 10.1097/01.blo.0000096825.67494.e3 14646713

[12] Long M, Rack HJ (1998). Titanium alloys in total joint replacement–a materials science perspective. Biomaterials.

[13] Tang X, Ahmed T, Rack HJ (2000). Phase transformations in Ti-Nb-Ta and Ti-Nb-Ta-Zr alloys. J Mater Sci..

[14] Niinomi M (1998). Mechanical properties of biomedical titanium alloys. Mater Sci Eng A.

[15] Mishra AK, Davidson JA, Poggie RA, Kovacs P, Fitzgerald TJ (1996). Mechanical and Tribological Properties and Biocompatibility of Diffusion Hardened Ti–13Nb–13Zr – A New Titanium Alloy for Surgical Implants. ASTM.

[16] Samuel S (2010). Corrosion resistance and *in vitro* response of laser-deposited Ti-Nb-Zr-Ta alloys for orthopedic implant applications. J Biomed Mater Res A.

[17] Fadl-allah SA, Mohsen Q (2010). Characterization of native and anodic oxide films formed on commercial pure titanium using electrochemical properties and morphology techniques. Appl Surf Sci..

[18] Casaletto MP (2001). Surface studies of *in vitro* biocompatibility of titanium oxide coatings. Appl. Surf. Sci..

[19] Yang B, Uchida M, Kim HM, Zhang X, Kokubo T (2004). Preparation of bioactive titanium metal via anodic oxidation treatment. Biomaterials.

[20] Raja KS, Misra M, Paramguru K (2005). Formation of self-ordered nano-tubular structure of anodic oxide layer on titanium. Electrochim Acta.

[21] Li P (1994). The role of hydrated silica, titania, and alumina in inducing apatite on implants. J Biomed Mater Res.

[22] Kamitakahara M, Kawashita M, Miyata N, Kokubo T, Nakamura T (2003). Apatite formation on CaO-free polydimethylsiloxane (PDMS)-TiO2 hybrids. J Mater Sci Mater Med.

[23] Green SA, Ripley MJ (1984). Chronic osteomyelitis in pin tracks. J Bone Joint Surg Am.

[24] Darouiche RO (2004). Treatment of infections associated with surgical implants. N Engl J Med.

[25] Crawford GA, Chawla N, Das K, Bose S, Bandyopadhyay A (2007). Microstructure and deformation behavior of biocompatible TiO2 nanotubes on titanium substrate. Acta Biomater.

[26] Cui L (2007). Repair of cranial bone defects with adipose derived stem cells and coral scaffold in a canine model. Biomaterials.

[27] Das K, Bose S, Bandyopadhyay A (2009). TiO_2_ nanotubes on Ti: Influence of nanoscale morphology on bone cell-materials interaction. J Biomed Mater Res A.

[28] Costa F, Carvalho IF, Montelaro RC, Gomes P, Martins MC (2011). Covalent immobilization of antimicrobial peptides (AMPs) onto biomaterial surfaces. Acta Biomater.

[29] Dey T, Roy P, Fabry B, Schmuki P (2011). Anodic mesoporous TiO2 layer on Ti for enhanced formation of biomimetic hydroxyapatite. Acta Biomater.

[30] Zhao L, Chu PK, Zhang Y, Wu Z (2009). Antibacterial coatings on titanium implants. J Biomed Mater Res B Appl Biomater.

[31] Clarke M, Bennett M, Littlewood T (2007). Cell death in the cardiovascular system. Heart.

[32] Murakami A (2012). Antimicrobial and osteogenic properties of a hydrophilic-modified nanoscale hydroxyapatite coating on titanium. Nanomedicine.

[33] Saji VS, Choe HC, Brantley WA (2009). An electrochemical study on self-ordered nanoporous and nanotubular oxide on Ti-35Nb-5Ta-7Zr alloy for biomedical applications. Acta Biomater.

[34] Rho JY, Kuhn-Spearing L, Zioupos P (1998). Mechanical properties and the hierarchical structure of bone. Med Eng Phys.

[35] Poinern GEJ (2012). Biocompatibility of Synthesised Nano-Porous Anodic Aluminium Oxide Membranes for Use as a Cell Culture Substrate for Madin-Darby Canine KidneysCells: A Preliminary Study. J Tissue Sci Eng..

[36] Hao Y, Li S, Han X, Hao Y, Ai H (2013). Effects of the surface characteristics of nanoporous titanium oxide films on Ti-24Nb-4Zr-8Sn alloy on the initial adhesion of osteoblast-like MG-63 cells. Exp Ther Med.

[37] Zhao C, Zhang X, Cao P (2011). Mechanical and electrochemical characterization of Ti–12Mo–5Zr alloy for biomedical application. J Alloy Compd..

[38] Macak JM, Tsuchiya H, Schmuki P (2005). High-aspect-ratio TiO_2_ nanotubes by anodization of titanium. Angew Chem Int Ed Engl.

[39] Park J, Bauer S, von der Mark K, Schmuki P (2007). Nanosize and vitality: TiO_2_ nanotube diameter directs cell fate. Nano Lett.

[40] Oh S (2009). Stem cell fate dictated solely by altered nanotube dimension. Proc Natl Acad Sci USA.

[41] Liu X, Chu PK, Ding C (2004). Surface modification of titanium, titanium alloys, and related materials for biomedical applications. Mater Sci Eng R..

[42] Pauksch L (2014). Biocompatibility of silver nanoparticles and silver ions in primary human mesenchymal stem cells and osteoblasts. Acta Biomater.

[43] Agarwal A (2010). Surfaces modified with nanometer-thick silver-impregnated polymeric films that kill bacteria but support growth of mammalian cells. Biomaterials.

[44] Jiang P, Liang J, Lin C (2013). Construction of micro–nano network structure on titanium surface for improving bioactivity. Appl Surf Sci..

[45] Wei D, Zhou Y, Yang C (2009). Structure, cell response and biomimetic apatite induction of gradient TiO2-based/nano-scale hydrophilic amorphous titanium oxide containing Ca composite coatings before and after crystallization. Colloids Surf B Biointerfaces.

[46] Bayatia MR (2010). Investigation on hydrophilicity of micro-arc oxidized TiO_2_ nano/micro-porous layers. Electrochim Acta..

[47] Geetha M, Mudalki KU, Gogia AK, Asokamani R, Raj B (2004). Influence of microstructure and alloying elements on corrosion behavior of Ti–13Nb–13Zr alloy. Corros. Sci..

[48] Collings, E.W. Advanced Structural Materials. *ASM, Metals Park*, **261** (1984).

[49] Antipov AI, Moiseev VN (1997). Coefficient of β-stabilization of titanium alloys. Met Sci Heat Treat..

[50] Chung CJ (2008). Photocatalytic TiO_2_ on copper alloy for antimicrobial purposes. Appl Catal B..

[51] Hashemzadeh F, Rahimi R, Gaffarinejad A (2013). Photocatalytic degradation of methylene blue and rhodamine B dyes by niobium oxide nanoparticles synthesized via hydrothermal method. International Journal of Applied Chemical Sciences Research..

[52] Fiz Raquel, Appel Linus, Mathur Sanjay (2013). Photoinduced Hydrophilicity and Photocatalytic Properties of Nb2O5Thin Films. Advanced Ceramic Coatings and Materials for Extreme Environments III.

[53] Chen HT (2010). Osteoblast growth behavior on micro-arc oxidized β-titanium alloy. Surf Coating Tech..

[54] Chen HT, Chung CJ, Yang TC, Tang CH, He JL (2013). Microscopic observations of osteoblast growth on micro-arc oxidized β titanium. Appl Surf Sci..

[55] Chung CJ (2009). Inactivation of Staphylococcus aureus and Escherichia coli under various light sources on photocatalytic titanium dioxide thin film. Surf Coating Tech..

[56] Chung CJ (2009). Mutifunctional arc ion plated TiO2 photocatalytic coatings with improved wear and corrosion protection. Surf Coating Tech..

[57] Seah KHW, Thampuran R, Chen X, Teoh SH (1995). A comparaison between the corrosion behavior of sintered and unsintered porous titanium. Corro. Sci..

[58] Aparicio C, Gil FJ, Fonseca C, Barbosa M, Planell JA (2003). Corrosion behaviour of commercially pure titanium shot blasted with different materials and sizes of shot particles for dental implant applications. Biomaterials.

[59] Ong KG, Varghese OK, Mor GK, Grimes CA (2005). Numerical simulation of light propagation through highly-ordered titania nanotube arrays: dimension optimization for improved photoabsorption. J Nanosci Nanotechnol.

[60] Karpagavalli R, Zhou A, Chellamuthu P, Nguyen K (2007). Corrosion behavior and biocompatibility of nanostructured TiO_2_ film on Ti6Al4V. J Biomed Mater Res A.

[61] Sur I, Cam D, Kahraman M, Baysal A, Culha M (2010). Interaction of multi-functional silver nanoparticles with living cells. Nanotechnology.

[62] Sur I, Altunbek M, Kahraman M, Culha M (2012). The influence of the surface chemistry of silver nanoparticles on cell death. Nanotechnology.

[63] Kearns DB (2010). A field guide to bacterial swarming motility. Nat Rev Microbiol.

[64] Roberts AE, Maddocks SE, Cooper RA (2015). Manuka honey reduces the motility of Pseudomonas aeruginosa by suppression of flagella-associated genes. J Antimicrob Chemother.

[65] Pollitt EJG, Diggle SP (2017). Defining motility in the Staphylococci. Cell Mol Life Sci.

[66] Schierholz JM, Lucas LJ, Rump A, Pulverer G (1998). Efficacy of silver-coated medical devices. J Hosp Infect.

[67] Martinez-Gutierrez F (2013). Anti-biofilm activity of silver nanoparticles against different microorganisms. Biofouling.

[68] Standard Test Methods for Tension Testing of Metallic Materials. *ASTM* E8 (2013).

